# Using mechanistic model-based inference to understand and project epidemic dynamics with time-varying contact and vaccination rates

**DOI:** 10.1038/s41598-022-25018-3

**Published:** 2022-11-28

**Authors:** Michael J. Plank, Shaun C. Hendy, Rachelle N. Binny, Giorgia Vattiato, Audrey Lustig, Oliver J. Maclaren

**Affiliations:** 1 School of Mathematics and Statistics, University of Canterbury, Christchurch, New Zealand; 2 Department of Physics, University of Auckland, Auckland, New Zealand; 3 Manaaki Whenua, Lincoln, New Zealand; 4 Department of Engineering Science, University of Auckland, Auckland, New Zealand

**Keywords:** Infectious diseases, Applied mathematics, Statistics

## Abstract

Epidemiological models range in complexity from relatively simple statistical models that make minimal assumptions about the variables driving epidemic dynamics to more mechanistic models that include effects such as vaccine-derived and infection-derived immunity, population structure and heterogeneity. The former are often fitted to data in real-time and used for short-term forecasting, while the latter are more suitable for comparing longer-term scenarios under differing assumptions about control measures or other factors. Here, we present a mechanistic model of intermediate complexity that can be fitted to data in real-time but is also suitable for investigating longer-term dynamics. Our approach provides a bridge between primarily empirical approaches to forecasting and assumption-driven scenario models. The model was developed as a policy advice tool for New Zealand’s 2021 outbreak of the Delta variant of SARS-CoV-2 and includes the effects of age structure, non-pharmaceutical interventions, and the ongoing vaccine rollout occurring during the time period studied. We use an approximate Bayesian computation approach to infer the time-varying transmission coefficient from real-time data on reported cases. We then compare projections of the model with future, out-of-sample data. We find that this approach produces a good fit with in-sample data and reasonable forward projections given the inherent limitations of predicting epidemic dynamics during periods of rapidly changing policy and behaviour. Results from the model helped inform the New Zealand Government’s policy response throughout the outbreak.

## Introduction

Between March 2020 and March 2022, New Zealand used strong border controls to minimise the importation of SARS-CoV-2 into the community from overseas. This enabled it to eliminate community transmission of the virus for long periods of time^1,2^. Overall incidence of COVID-19 was kept very low until vaccines became widely available, with a total of approximately 3,000 confirmed cases (0.06% of the population) up to August 2021. New Zealand’s roll out of the Pfizer/BioNTech BNT162b2 vaccine began in February 2021, initially targeted to frontline border workers and high-risk groups^3^. However, due to restricted supply of the vaccine, mass vaccination in the general population did not begin until July 2021.

On 17 August 2021, a new community case of COVID-19 was detected with no known link to the border. The outbreak was subsequently confirmed by whole genome sequencing to be the B.1.617.2 (Delta) variant and was linked to an ongoing Delta outbreak in the Australian state of New South Wales^4^. At this time, 32% of the population had received at least one dose of the vaccine, 19% had received two doses, and around 300,000 doses (6% of the population) were being administered per week. The government immediately moved the country to Alert Level 4, the most stringent level of restrictions, including stay-at-home orders with exceptions for essential workers only. This dramatically reduced transmission of the virus, with new cases falling to around 20 per day in September 2021 after an initial peak of 80 per day. However, unlike previous border-related incursions^2,5,6^, the virus was not eliminated and daily case numbers started to grow as restrictions were relaxed. The government formally shifted away from the elimination strategy on 4 October 2021^7,8^.

In the period between August 2021 and January 2022, the proportion of the population with two doses of the vaccine increased from 19 to 76% (corresponding to 93% of the eligible population over 12 years old). During the same period, restrictions and closures were progressively relaxed, while an intensive test-trace-isolate-quarantine system was used to suppress transmission. Continued strong border controls minimised importation of SARS-CoV-2 and the outbreak was largely contained to the Auckland region with travel restrictions^4^. Locally acquired cases peaked at around 200 per day in mid-November 2021 before declining steadily to 25 per day in January 2022, with a total of 11,523 confirmed community cases between 17 August 2021 and 23 January 2022. The number of hospitalised cases peaked at 93 on 29 November 2021 and there were 30 deaths. The outbreak disproportionately affected Māori and Pacific people^9^, who accounted for around 45% and 29% of cases and 39% and 34% of hospitalisations respectively, despite only making up 12% and 16% of the Auckland population. On 23 January 2022, the first community cases of the B.1.1.529 (Omicron) variant were reported and led to a rapidly growing outbreak, which required a different strategy and different set of modelling assumptions^10^.

Mathematical modelling has been an important tool for understanding and responding to the pandemic. A major challenge for mathematical models is accounting for changes in the amount of mixing between individuals over time^11^. These changes may occur as a result of government policy and spontaneous behaviour change in response to perceived risk or other factors. The timing, direction, and magnitude of these changes is usually difficult to predict in advance^12^. However, their effect can be estimated retrospectively by fitting models to data on reported cases, deaths or other epidemiological data streams^13–16^. This may help parameterise subsequent models and quantify uncertainty in model outputs.

Simulation-based inference methods, such as approximate Bayesian computation (ABC)^17^, are a common approach to parameter inference in mechanistic epidemic models^18^. At their simplest, these provide a way to perform inference on fixed epidemiological parameters, such as the basic reproduction number ${R}_{0}$. In reality, transmission rates are liable to vary over time due to changes in government policy, public health interventions and individual behaviour. In principle, simulation-based inference approaches extend to these more complicated settings; however, naïve ABC often performs poorly in high dimensions^17^, motivating extensions to ABC for more complex models^19–21^. A different approach is to empirically estimate the time-varying effective reproduction number in real-time while requiring minimal modelling assumptions^22–25^. These more empirically-oriented approaches can be used for nowcasting or forecasting. However, such methods typically cannot offer insights into the mechanisms driving changes in the epidemic dynamics, particularly the relative contributions of changes in contact rates and changes in population susceptibility, whether as a result of vaccination or immunity from prior infection.

In this paper, we combine a stochastic mechanistic epidemic model with an ABC sequential Monte Carlo (ABC-SMC) method^26–28^ to estimate and interpret the time-varying effective reproduction number during New Zealand’s 2021 Delta outbreak. Our mechanistic model allows us to decompose the contributions to variations in the effective reproduction number from the ongoing vaccine rollout, relaxation of control measures and accumulation of infection-derived immunity. The model we use builds on a previously published age-structured stochastic model^29^, generalised to include dynamic vaccination rates and a time-dependent control function, representing changing contact rates in the community. Vaccine coverage is based on a combination of data on vaccines administered and projected future uptake. We use ABC-SMC to estimate the time-varying control function from data on new daily cases. This allows the model to be fitted to data in real time and to project epidemic dynamics and health impacts forward in time under given assumptions about future contact rates. The inference algorithm could readily be applied to other epidemiological models as it takes as input a user-supplied routine to simulate the forward model under a given set of parameters and compute the distance function.

Our approach provides a bridge between statistical nowcasting/forecasting models and longer-term scenario models, producing medium-term projections and enabling a data-driven approach to epidemic scenario modelling. The model was used in real time throughout New Zealand’s Delta outbreak to provide situational awareness and epidemic projections. These results informed policy advice via the cross-agency COVID-19 Modelling Government Steering Group.

## Methods

We modelled transmission of SARS-CoV-2 using a discrete time, stochastic branching process model^30^. We build on the age-structured model of^29^, which assumed that susceptibility to infection, severe disease and death are age- and vaccination-status-dependent, and that contact rates between age groups are determined by a contact matrix^31^. The model assumed that the susceptible population was homogenous within each age and vaccination status group. However, it explicitly tracks infected individuals, which allowed us to model the test-trace-isolate-quarantine system for symptom-triggered testing and contact tracing. The model assumed that symptomatic individuals have a 45% probability of being tested and returning a positive result, and that contacts of confirmed cases have a 70% probability of being traced and quarantined (see Supplementary Material Sects. 1.1 and 1.2). Here, we generalised the model to include time-varying vaccine coverage and contact rates. The model is comparable in structure and assumptions to other models of the Delta variant of SARS-CoV-2 used for policy advice internationally^12,32–35^. MATLAB code to run the model and reproduce the results is available at https://github.com/michaelplanknz/model-inference-covid19-nz2021.

### Vaccine effectiveness and coverage

The Pfizer/BioNTech BNT162b2 vaccine accounts for the overwhelming majority of vaccine doses administered in New Zealand. Vaccine effectiveness was characterised in the model by four parameters: effectiveness against infection (${e}_{I}$), effectiveness against symptomatic disease in breakthrough infections (${e}_{S}$); reduction in transmission in breakthrough infections (${e}_{T}$), and effectiveness against severe disease or death in breakthrough infections (${e}_{D}$) after either one or two doses of the Pfizer vaccine against the Delta variant. Vaccine effectiveness parameters were based on estimates available in October 2021 from^36,37^ – see Supplementary Table S1 and^29^ for a more detailed literature review. Vaccination was assumed to provide a fixed reduction in risk across all age groups.

The proportion of people in each age group who have received one or two doses of the vaccine was time-varying based on vaccinations administered up to that date, as well as Ministry of Health data on future bookings. Since the outbreak was largely contained to the Auckland region (86% of all reported cases between 17 August 2021 and 23 January 2022), we used vaccination data for the Auckland metropolitan area (Auckland, Waitematā and Counties Manukau District Health Boards). In addition, we assumed that everyone who had received their first dose or had booked an appointment for it would eventually receive their second dose. For those who had their first dose but did not have an appointment booked for their second dose, the time of the second dose was assumed to be uniformly distributed in the 8 week period following the date at which the data on vaccines administered was accessed. Population denominators for each age band were taken to be the estimated resident population (ERP) according to StatsNZ (see Supplementary Table S1). Note that model vaccine coverage was lower than official Ministry of Health statistics because the latter use the health service utilisation (HSU) population, which is typically smaller than the ERP, as denominators. To model the delay in the immune response to vaccination, all vaccine doses were assumed to take effect 14 days after being administered.

### Age-structured transmission model

Transmission between age groups was described by a next generation matrix, whose $\left(i,j\right)$ element is defined to be the expected number of secondary infections in age group $i$ caused by an infected individual in age group $j$ in the absence of control measures and given a fully susceptible population. This was modelled as the product of the average number of contacts an individual in age group $j$ has with someone in age group $i$ during their infectious period, the probability of transmission per contact, the relative infectiousness of individuals in age group $j$, and the relative susceptibility of individuals in age group $i$ based on the approach of^29^ (see Supplementary Material Sect. 1.3). The average number of contacts between individuals in given age groups was based on age-structured contact matrices estimated by^31^, adapted for the New Zealand population by^29^. The next generation matrix was normalised to give a basic reproduction number of ${R}_{0}=6$ representing the highly transmissible Delta variant of SARS-CoV-2.

Each age group $j$ has three susceptible compartments representing people who have not been previously infected and have received 0, 1 or 2 vaccine doses at time $t$ (see Supplementary Figure S1). Vaccinated individuals have their probability of infection given exposure reduced by ${e}_{I}.$ This is known as a leaky vaccine model as opposed to an all-or-nothing vaccine model, where a proportion ${e}_{I}$ of vaccinated individuals are completely immunised and a proportion $1-{e}_{I}$ are completely susceptible^34^. Reality may be somewhere between these idealised models (i.e. there may be some individual heterogeneity in the level of protection provided by the vaccine but not as extreme as all-or-nothing). The all-or-nothing and the leaky vaccine model behave similarly when the proportion of the population with immunity from prior infection is relatively small.

We ignored waning of vaccine-derived immunity and infection-derived immunity. In reality, immunity wanes with time although for the Delta variant this can be counteracted to a large extent by a third (booster) dose of the vaccine^38,39^. In New Zealand, a third dose was offered to all adults starting in December 2021, initially with a minimum 6 month interval between the second and third dose. Here, we only modelled the period up to mid January 2022, when the majority of vaccinated New Zealanders were still within 3 months of their second dose, so the effects of waning are likely to be relatively minor over the time frame considered. Therefore, we did not attempt to model the dynamics of waning and boosting of immunity, although we note that this can be accommodated within this model framework^10^. The effects of seasonality in transmission rates were ignored.

### Disease severity

The risk of hospitalisation following infection for unvaccinated individuals was based on the estimates of^40^ in five year age bands, adjusted for the increased severity of the Delta variant with an odds ratio of 2.26^41^ (see Supplementary Table S1). To model the Auckland Delta outbreak, we increased risk of hospitalisation by a further odds ratio of 2 to give a better match with observed hospitalisation rates. This is partly explained by the fact that the outbreak disproportionately affected Māori and Pacific populations^9^, who are known to have a higher risk of hospitalisation with COVID-19^42^. The age-specific infection fatality ratio (IFR) was also based on the estimates of^40^. We did not apply an adjustment to IFR for the Delta variant. Although some studies have found an increased risk of fatality with Delta relative to previous variants ^43^, this may be offset somewhat by improved treatments for COVID-19. For more details on the hospitalisation and fatality submodel see Supplementary Material Sect. 1.4.

### Time-dependent transmission and parameter inference

For simplicity, we assumed that vaccination, case isolation, and public health restrictions act independently to provide multiplicative reductions in ${R}_{eff}$. It is possible that vaccination and public health restrictions do not act independently if, for example, vaccination rates differ for those who are able to work from home during periods of restrictions. The effect of different Alert Level restrictions or different levels of the COVID-19 Protection Framework (see Table 1) were unknown a priori and likely changing over time. To model this, we introduced a time-varying control factor $C(t)$, which acts multiplicatively on the transmission rate at time $t.$ This approach assumed that restrictions only change the next generation matrix $M$ by an overall multiplicative factor. This is not completely realistic as contact rates between different age groups are likely to respond differently, but was a necessary model assumption in the absence of specific data to parameterise a time-dependent contact matrix.

**Table 1:** Summary timeline of key restrictions and public health measures in response to the outbreak of the B.1.617.2 (Delta) variant in Auckland from August 2021.

Dates	Setting	Key changes to restrictions
Up to 17/08/21	Alert Level 1	Masks required on public transport and mandatory record keeping. No other restrictions on domestic activity
18/08/21 – 21/09/21	Alert Level 4	Strict stay-at-home orders for non-essential workers with limited expcetions
22/09/21 – 05/10/21	Alert Level 3	Reopening of contactless businesses and limited non-essential workplaces and services
06/10/21 – 09/11/21	Alert Level 3, Step 1	Small outdoor social gatherings and recreation allowed. Early childhood education reopens
10/11/21 – 02/12/21	Alert Level 3, Step 2	Retail and public facilities reopen with mandatory masks
03/12/21 – 30/12/21	COVID-19 Protection Framework, Red	All businesses, services and facilities may open provided vaccine passes are used. Public gatherings limited to 100 and capacity limits based on one-metre distancing. Masks required in many public settings. Working from home encouraged
31/12/21 onwards	COVID-19 Protection Framework, Orange	Gathering size and capacity limits removed provided vaccine passes are used

Other parts of New Zealand were subject to different restrictions at different times. However, travel restrictions were used to largely contain the outbreak to the Auckland region for the majority of the period considered so we focus on the Auckland restrictions here.

Although public health measures are likely to be strongly correlated with $C(t)$, other factors will influence contact rates such as compliance levels and behavioural changes in response to perceived risk. Rather than explicitly tying changes in $C(t)$ to changes in public health restrictions, we allowed $C(t)$ to change in weekly time periods by defining $C\left(t\right)$ to be a piecewise constant function: $C\left(t\right)={C}_{k}$ when ${t}_{k-1}\le t<{t}_{k}$, with ${t}_{1}$ corresponding to 18 August 2021 (the first day of Alert Level 4), ${t}_{2},\dots {t}_{K-1}$ increasing in 7-day increments, and ${t}_{K}$ corresponding to the last day of the simulation. The number of time periods was chosen such that there was a minimum of 21 days between ${t}_{K-1}$ and the final day for which data was available. This ensured sufficient data was available to estimate the value of $C\left(t\right)$ in the final time period, allowing for the time lag between changes in $C(t)$ and its effect in reported case numbers.

We estimated the values of ${C}_{k}$ by fitting model output to data on new daily cases, using an approximate Bayesian computation (ABC) approach. We used a log-transformed Gaussian random field as the prior for $C=[{C}_{1},{C}_{2},\dots {C}_{K}$], which encodes correlation structure between neighbouring control function values. We sampled from the prior by generating random deviates ${\theta }_{k}$ from a standard multivariate normal distribution and transforming them according to $C=\mathrm{exp}(m+\theta L)$ where ${m}_{k}$ is the mean of $\mathrm{ln}({C}_{k})$, $L$ is the Cholesky decomposition of the covariance matrix ${\Sigma }_{0}=DAD$, $D$ is a diagonal matrix of standard deviations of $\mathrm{ln}({C}_{k})$, and ${A}_{kl}=\mathrm{exp}\left(-{\left(k-l\right)}^{2}/2{L}^{2}\right)$ is the correlation matrix. This matrix has squared exponential form with correlation length $L=2$, reflecting an a priori assumption that the control function is temporally autocorrelated with a characteristic timescale of 2 weeks. The parameters ${m}_{k}$ and ${D}_{kk}$ are related to the mean ${\mu }_{k}$ and variance ${\sigma }_{k}^{2}$ of ${C}_{k}$ via the usual log-normal distribution parameterisation. For time periods $k=2,\dots K$ we set ${\mu }_{k}=0.5$ and ${\sigma }_{k}=0.5$. For the first time period ($k=1$), we set ${\mu }_{k}=1$ and ${\sigma }_{k}=0.2$ as transmission is expected to be higher during this period prior to outbreak detection and the move to Alert Level 4. We set the off-diagonal elements in the first row and column of $A$ to be zero so that ${C}_{1}$ is independent of $[{C}_{2},\dots {C}_{K}$]. Together these choices represent a relatively uninformative prior for the transmission rates during the period when public health restrictions were in place (see Supplementary Figure S2).

Given the above prior, we computed an approximate posterior distribution for $\theta$ by conditioning on data on the number of new daily community cases of COVID-19 reported by the Ministry of Health. We did this using an ABC method with sequential Monte Carlo (ABC-SMC) based closely on the replenishment algorithm of^26^ – see Algorithm 1. Like all ABC methods, this involves drawing proposal values for the parameters $\theta$ from the prior $\pi (\theta )$ defind above and running the model to generate a simulated dataset $x \sim \pi (.|{\theta })$, in this case representing a time series of new daily cases. These are compared to the real data ${x}^{data}$ using a distance function $\Delta \left(x,{x}^{data}\right)$ (see below) and the proposal values satisfying $\Delta \left(x,{x}^{data}\right)<{\epsilon }_{0}$ for some threshold ${\epsilon }_{0}$ are retained. This is known as an ABC rejection step^17^ and provides a sample $\left\{{\theta }_{i}\right\}$ from the distribution $\pi \left(\theta \right|\Delta \left(x,{x}^{data}\right)<{\epsilon }_{0})$. However, naive ABC rejection is typically very inefficient, especially with a high-dimensional parameter space. ABC-SMC improves efficiency by propagating the set of particles $\left\{{\theta }_{i}\right\}$ through a series of intermediate distributions $\pi \left(\theta \right|\Delta \left(x,{x}^{data}\right)<{\epsilon }_{t})$ with ${\epsilon }_{0}\ge \dots \ge {\epsilon }_{T}$. The replenishment algorithm does this by retaining a proportion $\alpha$ of the previous set of particles $\left\{{\theta }_{i}\right\}$ with the smallest values of $\Delta \left(x,{x}^{data}\right)$ and maintaining a sample of size $N$ by resampling from the retained $\left\{{\theta }_{i}\right\}$ and perturbing each resampled particle ${R}_{t}$ times via a MCMC kernel. To maintain sample diversity, the number of MCMC iterations ${R}_{t}$ is chosen adaptively in terms of the MCMC acceptance rate ${p}_{acc}$ (see line 29 of Algorithm 1) such that there is a probability $1-c$ that all resampled particles are moved at least once^20,26^, where $c$ is a tuning parameter. This process is repeated until all $N$ particles meet the specified distance threshold ${\epsilon }_{T}$, or the MCMC acceptance rate drops below a specified minimum value ${f}_{acc}$.

We found this method produced similar results to the ABC-SMC algorithm of^27^, but was more computationally efficient, required less hyperparameter tuning, and avoided the need to weight particles which tended to cause problems with sample degeneracy. In addition, pre-calculating the Metropolis–Hastings ratio for a proposed value of $\theta$ prior to simulation of the model (see line 21 of Algorithm 1) reduced the amount of time spent sampling regions of parameter space where the prior probability density is low^26^.

**Figure:**
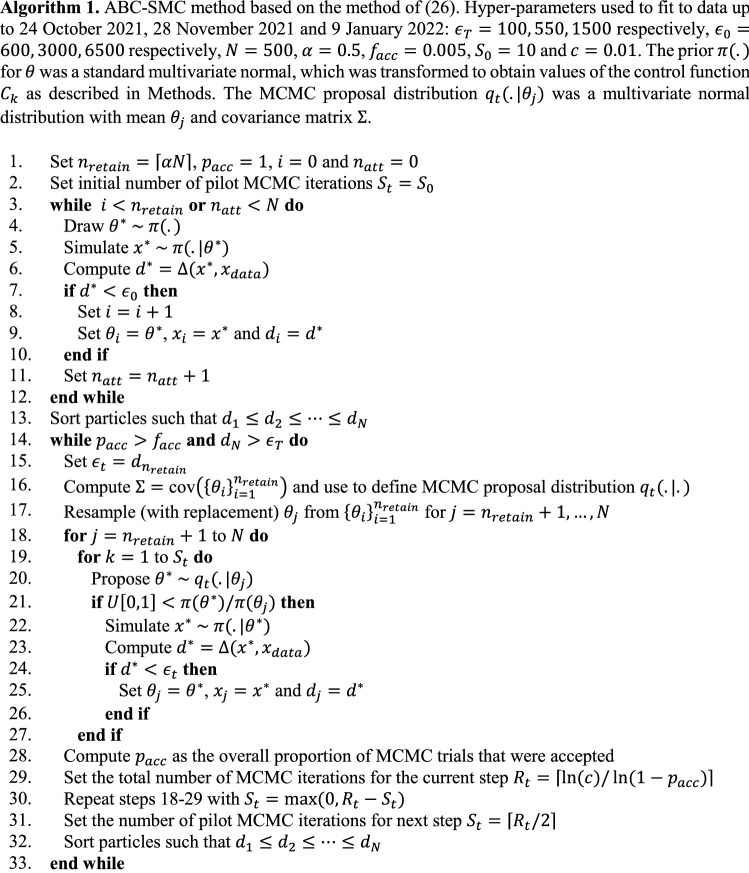

We defined the distance function on time series of daily reported cases $x=\left\{{x}_{i}\right\}$ to be the sum of squared errors on square-root transformed data:

$$\Delta \left(x,y\right)=\sum_{i}{\left(\sqrt{{x}_{i}}-\sqrt{{y}_{i}}\right)}^{2}$$

The square-root transformation is a variance-stabilising transformation suitable for data in which the variance increases with the mean, e.g. Poisson-like data^44^, and we found this appropriate here. However, using the sum of squared errors without the square-root transform (which is equivalent to using a Euclidean distance function) also led to very similar results. We generated $N=500$ particles using an acceptance rate of $\alpha =0.5$ per SMC step, minimum MCMC acceptance rate of ${f}_{acc}=0.005$, and tuning parameter $c=0.01$. When fitting to the complete dataset, we used a target threshold of ${\epsilon }_{T}=1200$, with smaller threshold values used when fitting to data from a subset of the time period (see Algorithm 1). Using a smaller target threshold produced a more accurate approximation to the true posterior at the expense of longer computation time. This combination of hyperparameters was found to give a reasonable combination of runtime and model fit, although the precise choice of hyperparameters did not have a major impact on results. Any simulations that exceeded the acceptance threshold before completion were terminated. This early rejection step required calculation of the partial sums of the distance function at each time step within the forward model, but was essential for computational efficiency. We additionally specified a maximum distance threshold ${\epsilon }_{0}=6500$ for the initial ABC rejection step. This is optional but allows early rejection of particles that have very poor fit to the data.

The data ${x}_{data}$ was defined to be the number of new reported community cases of COVID-19 on each day between 17 August 2021 and a specified cut-off date, using the *Lab confirmed date* field in EpiSurv (or *Date reported* field if *Lab confirmed date* is missing). Cases in recent international arrivals detected in managed isolation and quarantine were excluded.

### Projections of epidemic dynamics

Once the fitting routine was complete, we projected the model forwards in time by performing $n=2500$ independent model simulations with values of ${C}_{k}$ drawn from the estimated posterior distribution. For the final period ($t\ge {t}_{K-1}$), the control function $C(t)$ was assumed to remain constant at its final inferred value ${C}_{K}$. This corresponds to a projection of the epidemic dynamics under the assumption that future contact rates remain equal to the inferred contact rates in the most recent time period. These simulations were additionally filtered to retain the $n=500$ simulations with the smallest value of the distance function $\Delta (x,y)$. As well as improving model fit, this filtering step excluded stochastic realisations in which the epidemic went extinct during the period of very low reported case numbers in September 2021. Performing fewer simulations means more statistical noise in the results, whereas requiring more simulations increases the computational cost; retaining $n=500$ simulations was found to provide a reasonable balance between these considerations.

## Results and discussion

To investigate the fit of the model to data and the performance of model projections, we consider results fitted to data on daily reported cases from 17 August 2021 up to one of three different time points: 24 October 2021, 28 November 2021, and 9 January 2022. The first two dates correspond to times at which model results were communicated to policymakers to inform decisions about relaxing public health restrictions that were in force in Auckland at the time. The final date is towards the end of the Delta outbreak, when case numbers had fallen to very low levels and shortly before the B.1.1.529 (Omicron) variant became dominant in New Zealand.

**Figure 1:**
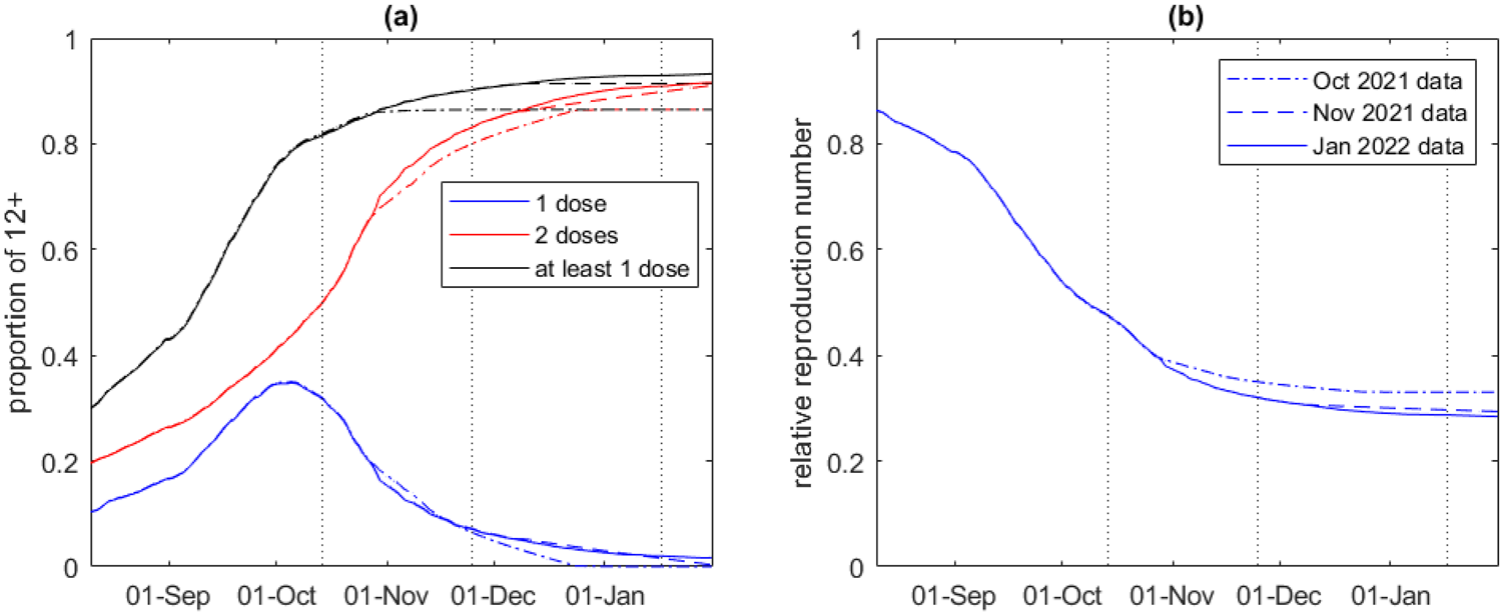
(**a**) Proportion of the Auckland population over 12 years old that had received 1 dose (blue), at least 1 dose (black), and 2 doses (red) over time. (**b**) Estimated effect of vaccination on the reproduction number over time. Vaccine coverage is calculated based on a combination of data on vaccines administered to date, data on future vaccination bookings, and projected future uptake of second doses (see *Vaccine effectiveness and coverage* section of Methods for details). Results are shown based on data accessed on three different dates (indicated by vertical dotted lines): 14 October 2021 (dash-dot), 25 November 2021 (dashed) and 17 January 2022 (solid).

Vaccine coverage in the Auckland region rose rapidly during the modelled time period, with the proportion of those aged over 12 years who had received two doses increasing from 20% in mid-August 2021 to 91% by mid-January 2022 (Fig. 1a). Due to the age prioritisation of the vaccine rollout, coverage was generally higher in older age groups (Supplementary Figure S3). Based on data available at the first fitted time point (15 October 2021), two-dose vaccine coverage was projected to reach around 87% of over-12-year-olds by mid December 2021 (Fig. 1a, dash-dot curves). In fact, vaccine coverage subsequently increased slightly faster and to a higher level than projected at this time, as seen in results based on data available at the second and third fitted points (Fig. 1a, dashed and solid curves respectively).

We used the model to calculate the effect of vaccine-derived immunity on the reproduction number, independently of other effects (Fig. 1b). At the start of the outbreak, the model estimated that vaccination was reducing the reproduction number by around 14%. Vaccination was projected to eventually reduce the reproduction number by around 67% (according to data available on 14 October 2021) and 72% (according to data available on 17 January 2022).

**Figure 2:**
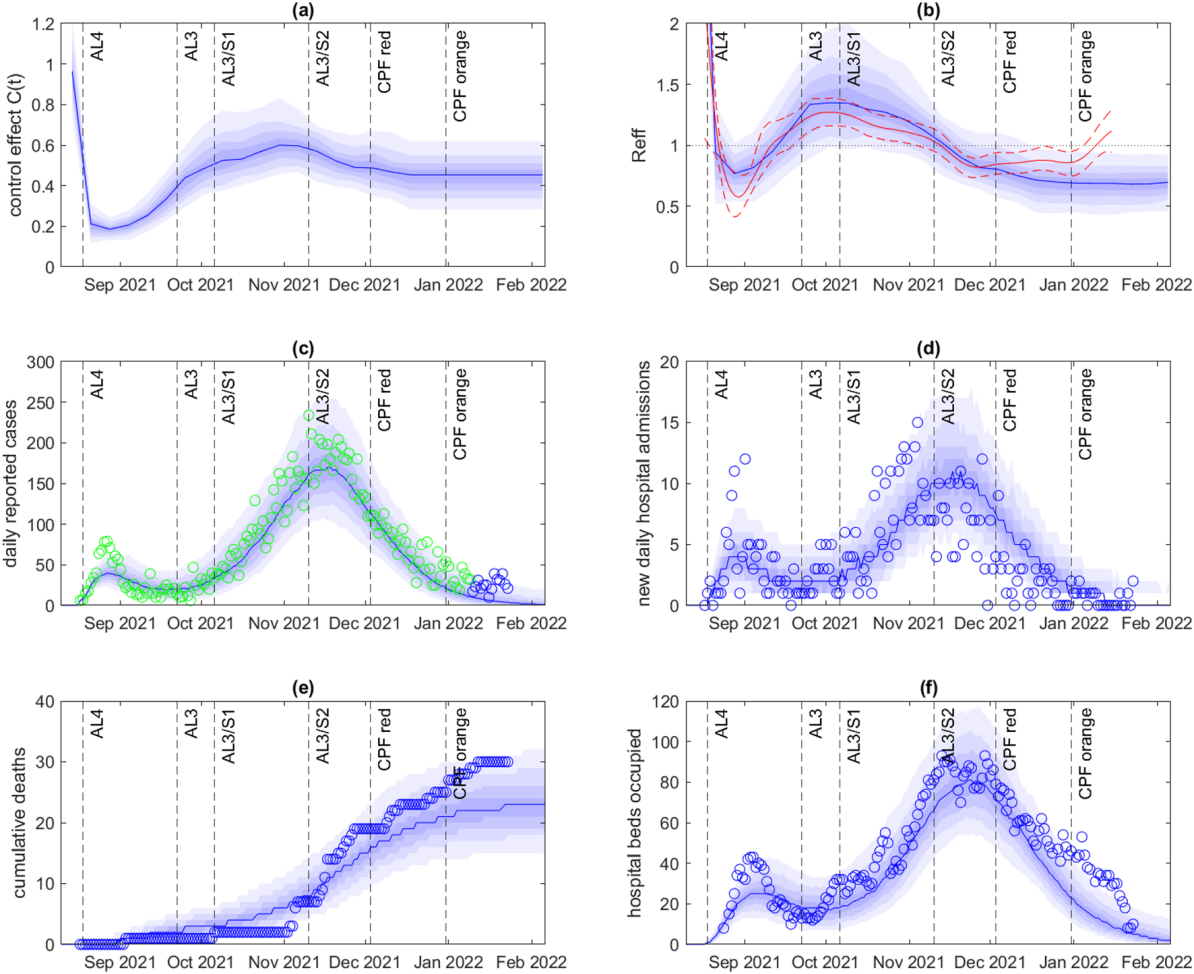
Model results fitted to data up to 9 January 2022. (**a**) Inferred control function $C(t)$; (**b**) Effective reproduction number ${R}_{eff}(t)$ calculated in the model (blue) based on time-varying control function $C(t)$ and vaccine-derived and infection-derived immunity, and independently estimated using the EpiNow2 package^23,45^ (median estimate red solid, 90% credible band red dashed); (**c**) Daily reported cases; (**d**) New daily hospital admission; (**e**) Cumulative deaths; (**f**) Hospital beds occupied. In (**c**), green points show data that was used to fit the control function shown in (**a**). In (**a**,**b**), the blue curve shows the median and the shaded blue bands show the 10th, 20th, 30th, 40th, 60th, 70th, 80th, and 90th percentiles of the estimated posterior distribution for $C(t)$. In (**c**–**f**), the blue curve shows the median and the shaded blue bands show the 10th, 20th, 30th, 40th, 60th, 70th, 80th, and 90th percentiles of the specified model output across the best-fitting $n=500$ out of 2500 model simulations, each with $C(t)$ sampled independently from the estimated posterior. Dashed vertical lines show the times of key changes to public health restrictions for the Auckland region (see Table 1).

After fitting to data on new daily cases up to 9 January 2022, the model provided a reasonable fit to the outbreak as a whole, although it slightly underestimated the number of cases towards the end of the time period (Fig. 2). The control function $C(t)$ (Fig. 2a), representing relative contact rates, was estimated to be around 1 prior to outbreak detection on 18 August 2021, corresponding to uncontrolled levels of transmission. Following outbreak detection and the move to Alert Level 4, the estimated control function dropped sharply. In the subsequent two months, the estimated control function steadily increased, suggesting progressively greater contact rates. Some of the observed increase in $C(t)$ may be explained by relaxation of restrictions to Alert Level 3 on 22 September and “Step 1” on 6 October (see Table 1). However, some of the increase in $C(t)$ pre-dates these changes suggesting a possible drop in compliance and/or voluntary behaviour change. It may also reflect the fact that, around this time, the outbreak was increasingly affecting parts of the population that were difficult to reach from a public health perspective and had limited ability to self-isolate – see also^4,8,9^. The estimated control function $C(t)$ was relatively steady from mid-October to mid-November, despite a relaxation of restrictions to “Step 2” on 10 November.

Surprisingly, the control function decreased around the time of the switch from the Alert Level system to the COVID-19 Protection Framework (CPF red in Fig. 2), a significant further relaxation of restrictions. It then remained at relatively low levels for the remainder of the time period, even after further relaxation to CPF orange. This may be partly explained by reduction in indoor mixing during the summer school holidays and Christmas period. However, as noted above, there are also some signs that the model is underfitting somewhat towards the end of the time period, with the data showing a slight uptick in case numbers while the model numbers continue to fall. This slight model misfit may be enough to keep the control function estimates too low and is likely due to a combination of the difficulty of sampling a relatively high-dimensional parameter space and the small number of cases during this time period.

Given an estimated control function $C\left(t\right)$, the corresponding time-varying effective reproduction number ${R}_{eff}(t)$ is a model output that depends on this control function as well as the effects of vaccine-derived and infection-derived immunity, and test-trace-isolate-quarantine (TTIQ) measures (Fig. 2b). For this outbreak, the contribution of infection-derived immunity was small as only a small proportion of the population was infected; our model-based ${R}_{eff}(t)$ hence has a similar shape to the control function, although is affected by the increasing levels of vaccine-derived immunity as shown in Fig. 1b. In contrast, the reproduction number estimated using the nowcasting package EpiNow2^23,45^ appears more sensitive to slight variations in cases and shows a small corresponding increase in ${R}_{eff}\left(t\right)$ after the shift to CPF red and a further increase after the shift to CPF orange. This highlights the potential impact of model (mis)specification or a poorly sampled parameter space on model-based projections and ${R}_{eff}\left(t\right)$ estimates. Despite these differences, however, the model reproduction number followed a reasonably similar trajectory to that estimated by EpiNow2 using daily cases up to 26 January 2022^45^ and the 90% credible bands overlapped for the duration of the simulation.

**Figure 3:**
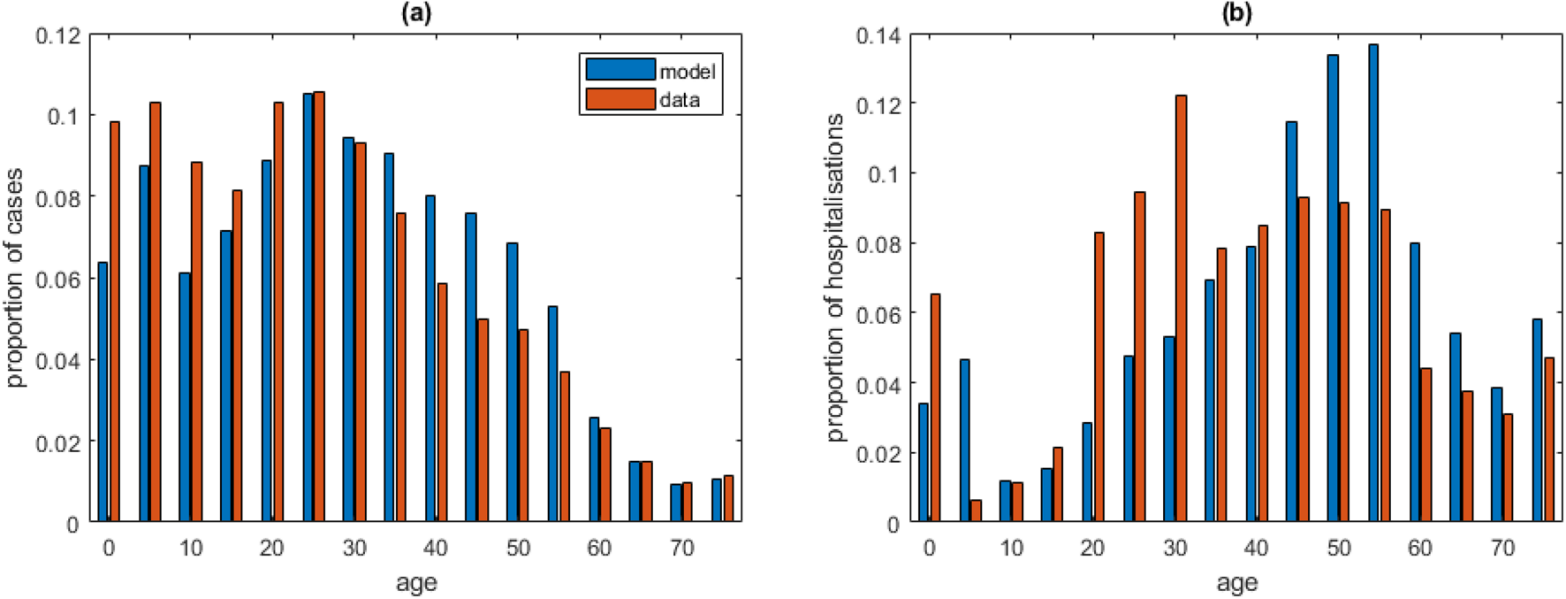
Age distribution of: (**a**) all cases; (**b**) hospitalised cases, comparing model (blue) with data (red) for New Zealand community cases reported between 17 August 2021 and 23 January 2022.

The model gave a reasonable match with the time series for hospital occupancy and deaths (Fig. 2d-f), which were not used in fitting the model. The age distribution of cases in the model was also qualitatively similar to the data (Fig. 3a), although the model underestimated the proportion of cases in the under 25 year age group and overestimated the proportion in the 35–60 year age group. The match to the age distribution of hospitalisations (Fig. 3b) was more variable, partly because the number of hospitalised cases was smaller. There have been more hospitalisations of under-5-year-olds than the model estimates (although a significant proportion of these may have been social admissions or for non-COVID-19 related treatment). The bulk of hospitalised cases were in the 20–60 year age group in both model and data.

**Figure 4:**
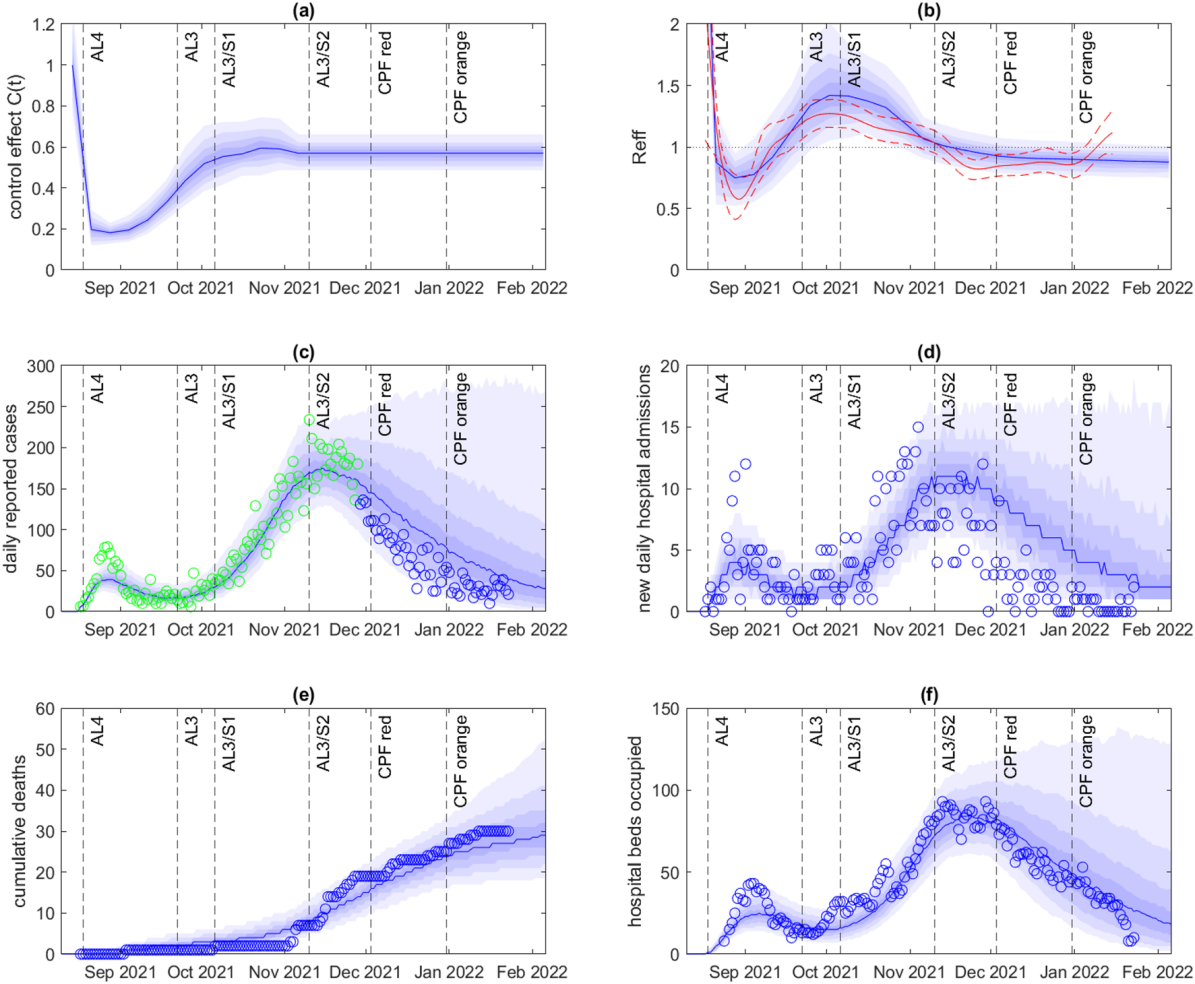
Model results fitted to data up to 28 November 2021. (**a**) Inferred control function $C(t)$; (**b**) Effective reproduction number ${R}_{eff}(t)$ calculated in the model (blue) based on time-varying control function $C(t)$, and vaccine-derived and infection-derived immunity, and independently estimated using the EpiNow2 package^23,45^ (median estimate red solid, 90% credible band red dashed); (**c**) Daily reported cases; (**d**) New daily hospital admission; (**e**) Cumulative deaths; (**f**) Hospital beds occupied. In (**c**), green points show data that was used to fit the control function shown in (**a**). In (**a**,**b**), the blue curve shows the median and the shaded blue bands show the 10th, 20th, 30th, 40th, 60th, 70th, 80th, and 90th percentiles of the estimated posterior distribution for $C(t)$. In (**c**–**f**), the blue curve shows the median and the shaded blue bands show the 10th, 20th, 30th, 40th, 60th, 70th, 80th, and 90th percentiles of the specified model output across the best-fitting $n=500$ out of 2500 model simulations, each with $C(t)$ sampled independently from the estimated posterior. Dashed vertical lines show the times of key changes to public health restrictions for the Auckland region (see Table 1).

We retrospectively investigated the results of fitting the model to data on cases in the first part of the outbreak and comparing with the future epidemic trajectory. These model results are not predictions because they do not take account of likely future changes in restrictions and/or behaviour. Rather, they are projections of the epidemic dynamics under the assumption that contact rates, as quantified by the control function $C(t)$, remain constant subsequent to the fitted time period. After fitting to case data up to 28 November 2021, approximately coinciding with the peak in reported cases, the fit of the model to in-sample data was good, but the model projection tended to overestimate the number of future cases in out-of-sample data (Fig. 4). In reality, cases fell faster following the peak than the model projected. This is related to the observation above that there was an unexpected decrease in the fitted control function in late November and early December when fitted to data through to January. However, hospital occupancy and deaths (Fig. 4e-f) remained close to the median projection and well within the 80% credible band for most of the simulated time period.

**Figure 5:**
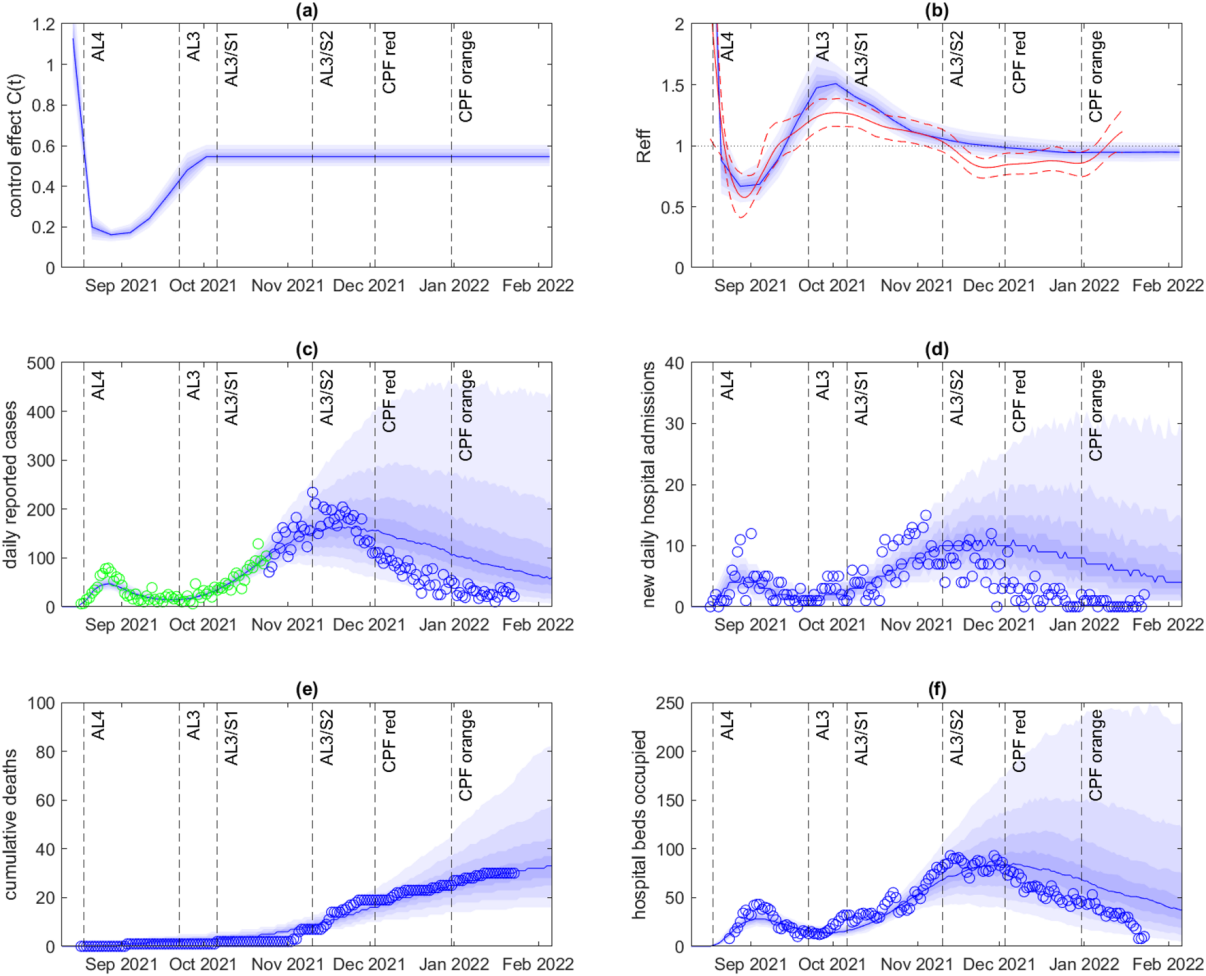
Model results fitted to data up to 24 October 2021. (**a**) Inferred control function $C(t)$; (**b**) Effective reproduction number ${R}_{eff}(t)$ calculated in the model (blue) based on time-varying control function $C(t)$, and vaccine-derived and infection-derived immunity, and independently estimated using the EpiNow2 package^23,45^ (median estimate red solid, 90% credible band red dashed); (**c**) Daily reported cases; (**d**) New daily hospital admission; (**e**) Cumulative deaths; (**f**) Hospital beds occupied. In (**c**), green points show data that was used to fit the control function shown in (**a**). In (a,b), the blue curve shows the median and the shaded blue bands show the 10th, 20th, 30th, 40th, 60th, 70th, 80th, and 90th percentiles of the estimated posterior distribution for $C(t)$. In (**c**–**f**), the blue curve shows the median and the shaded blue bands show the 10th, 20th, 30th, 40th, 60th, 70th, 80th, and 90th percentiles of the specified model output across the best-fitting $n=500$ out of 2500 model simulations, each with $C(t)$ sampled independently from the estimated posterior. Dashed vertical lines show the times of key changes to public health restrictions for the Auckland region (see Table 1).

After fitting to case data up to 24 October 2021, the model projection again provided a good fit to in-sample data, and also provided a good match with the size and timing of the subsequent peak in reported cases (Fig. 5). This is because a constant control function during late October and November 2021 was a reasonable approximation to the estimated control function fitted to subsequent data (Fig. 2a). This suggests that there was not a significant increase in contact rates during this period, despite the easing of restrictions to Alert Level 3, Step 2 on 10 November (see Table 1). The peak in cases was primarily driven by increasing vaccine-derived immunity, which was already built into the model in October 2021 via bookings for future vaccine appointments and projected uptake of second doses (see Fig. 1). The model again failed to predict the speed of the drop in cases following the peak in late November, although most observed variables remained within the 80% credible band for the duration of the simulation.

One general message to take away from these results is that, although the model can be used for short-term projections (< 1 month), it cannot generally be used to produce accurate medium- or long-term (> 1 month) predictions. The essential difficulty is that there is too much uncertainty in specifying the control function out of sample as it depends on potential future changes in public health policy and spontaneous behaviour change in response to perceived risk. The flexibility of the control function generally allows for good in-sample fitting (though even then it can be difficult to avoid slight underfitting), but this flexibility makes it difficult to specify for future scenarios given these many sources of uncertainty.

Nevertheless, our method for fitting the control function to data provides an avenue to better link short-term epidemic projections and long-term scenario modelling. For example, the retrospective estimate of the contact function under specific conditions may be useful for parameterising scenarios with different levels of public health measures or voluntary precautionary behaviour in place. It also ensures that models used for future scenario planning are capable of quantitatively explaining past observations. Linking retrospective estimates of the control function to correlates of contact rates such as mobility data or social survey data^16^^,^^11^ would also help integrate a range of data sources relating to behavioural change into epidemic models. We projected epidemic dynamics forward in time under the assumption that the control function remained at the current inferred level. It would also be possible to use the model to consider specific scenarios where the control function changed by a specified amount on certain dates, for example to model the anticipated effect of a potential future change in policy or behaviour. This can be useful to inform decisions about easing public health restrictions.

The model we used has the advantage of being a relatively simple representation of the epidemic dynamics, focusing on age and vaccination status as the most important variables affecting transmission and disease. Its relative simplicity means that the reasons the model produces a particular result can be explained and understood in terms of epidemiological mechanisms rather than being a black box. We found this to be advantageous for communicating the results and building an understanding the model and its limitations with health officials and policymakers. The model explicitly treats infected people as individuals rather than a homogeneous compartment, which enables the test-trace-isolate-quarantine system to be modelled at the individual level. However, treating the susceptible population as homogeneous (within each age and vaccination status group) means it does not have an unduly large number of unknown parameters and can reasonably be calibrated using routinely collected epidemiological data such as cases, hospitalisations and deaths. As we have demonstrated, the model can be fitted to epidemiological data streams to produce projections of epidemic dynamics in real time.

The model has a number of important limitations. The model assumes that relative contact rates between age groups are described according to the contact matrix estimated by^31^. In reality, contact rates may differ from these pre-pandemic estimates and are also likely to be affected by behavioural change and public health restrictions imposed in response to the outbreak. This could have a significant impact on model results due to the strong age-dependence in vaccine coverage and risk of severe disease. The assumed contact matrix provided a reasonable match with the age structure of confirmed cases in the outbreak. Nevertheless, social survey data collected in New Zealand under different conditions would help parameterise more realistic contact matrices and how these may respond to changing epidemiological situations^11,46^. The model ignores the effects of waning immunity, and so is only suitable for modelling an outbreak over a relatively short period of time, although we note the model framework can be generalised to include the effects of waning^10^. The model assumes that vaccine effectiveness is independent of age, which potentially ignores reduced effectiveness in older groups^47^. The model assumes that a stable fraction of infections are reported as cases. This is a reasonable assumption given that there was intensive case finding, source investigation, testing and contact tracing throughout the period modelled. However, it is possible that case ascertainment reduced as the outbreak spread into harder to reach parts of the population^4,8,9^. The model makes assumptions about the effectiveness of case isolation and contact quarantine. However, one advantage of fitting the model to data is that the main results are robust to these assumed parameters. For instance, if the effectiveness of these measures was consistently greater than assumed then, in order to preserve the fit with the data, the inferred value of the control function would have to be consistently larger and, therefore, the reproduction number and epidemiological time series output by the model would be similar. Model projections are sensitive to the assumed values of the vaccine effectiveness parameters because, given the low levels of infection-derived immunity, increasing vaccine coverage is the main mechanism causing the outbreak to peak. A sensitivity analysis of the model to vaccine effectiveness parameters was carried out by^29^.

The model ignores spatial and demographic heterogeneity in transmission and vaccination rates. The effects of this heterogeneity are likely to be more pronounced in a relatively small outbreak such as the one studied here. Some of this heterogeneity may be effectively subsumed into the fitted control function $C(t)$. In other words, $C(t)$ represents average contact rates in the subpopulation infected with the virus at time $t$, which may differ from contact rates the population as a whole. We have fitted a single time-varying parameter to data on daily cases only. It would be possible to fit to other data streams such as hospitalisations and deaths, which have the advantage of being less dependent on testing patterns. It would also be possible to treat other model parameters as targets for inference, for example probability of testing. However, care is needed to avoid overfitting and identifiability issues.

We have used a stochastic, individual-based model which is appropriate for a relatively small outbreak such that experienced in Auckland from August 2021. For a large outbreak involving a significant fraction of the population, this modelling approach wold be computationally inefficient. However, our ABC method for inferring the time-varying control function would naturally extend to more efficient deterministic compartment models. Epidemic models take a variety of forms depending on which variables and processes are included (e.g. age, vaccination, waning of immunity, population structure, testing and isolation). However, most epidemic models typically include a coefficient that scales the force of infection and is proportional to effective reproduction number at time $t$. If this coefficient is time-varying, it plays an equivalent role to the control function $C(t)$ in our framework and can be treated as a target for inference using our method. Future work modelling COVID-19 in New Zealand will need to include waning of vaccine-derived and infection-derived immunity, the effects of additional doses of the vaccine, and the effects of different variants of SARS-CoV-2.

## Supplementary Information

Supplementary Information.

## Data Availability

The datasets analysed during the current study and MATLAB code to run the model and reproduce the results are available in the Github repository, https://github.com/michaelplanknz/model-inference-covid19-nz2021.
