# Supplementary material for: Using mechanistic model-based inference to understand and project epidemic dynamics with time-varying contact and vaccination rates
Source: Sci Rep. 2022 Nov 28;12:20451. doi: 10.1038/s41598-022-25018-3 (PMC9702885; doi:10.1038/s41598-022-25018-3)
Supplement: Supplementary file 1 — Supplementary Information. [file 41598_2022_25018_MOESM1_ESM.docx]

**Using mechanistic model-based inference to understand and project epidemic dynamics with time-varying contact and vaccination rates Supplementary Material**

Contents

[1. Model specification 2](#_Toc118713152)

[1.1 Basic epidemiological parameters 2](#_Toc118713153)

[1.2 Test-trace-isolate-quarantine system model 2](#_Toc118713154)

[1.3 Age-structured transmission model 2](#_Toc118713155)

[1.4 Hospitalisation and fatality model 4](#_Toc118713156)

[1.5 Initial conditions 4](#_Toc118713157)

[2. Supplementary Figures 6](#_Toc118713158)

# Model specification

## Basic epidemiological parameters

We model transmission of SARS-CoV-2 using a stochastic age-structured branching process model. The model generalises that of (1) to include time-varying vaccine coverage and contact rates. Infected individuals are categorised as either clinical or subclinical, with the clinical fraction $p_{clin}$ increasing with age (see Supplementary Table S1). Subclinical individuals are assumed to be $\tau=50\%$ as infectious as clinical individuals (2-4). Clinical individuals are assigned a symptom onset time which is gamma distributed from exposure time with mean 5.5 days and s.d. 3.3 days (5). In the absence of interventions, we assume generation times are drawn from a Weibull distribution with mean 5.0 days and s.d. 1.9 days (6). There is at present conflicting evidence in the literature as to whether the Delta variant of SARS-CoV-2 has a shorter mean generation time or mean incubation period than older variants (7-11). Generation times in particular are difficult to empirically measure because this requires the infection times of both cases in a transmission pair. If infection times are unavailable but symptom onset dates are known, the serial interval can be used as a proxy for generation time. However, serial interval measurements contain more noise as they depend on both individuals’ incubation periods. In addition, for both generation times and serial intervals, realised values are affected by control interventions such as test, trace and isolate measures.

## Test-trace-isolate-quarantine system model

We assume that, for all infected individuals with clinical symptoms, the probability of becoming a confirmed case of COVID-19 as a result of symptom-triggered testing is $p_{test}=0.45$. We assume that confirmation occurs with an exponentially distributed delay from onset with mean 4 days. Although using an exponential distribution is a simplifying assumption, the coefficient of variation of the exponential distribution ($CV=1$) is similar to the coefficient of variation of observed data from the August 2021 outbreak on the time from symptom onset to reporting for cases designated as “sought healthcare” in EpiSurv ($CV=1.025$). The shape of the distribution is also approximately consistent with onset to reporting times from the August 2020 outbreak. Furthermore, we note that, for a given overall effect of TTIQ on $R_{eff}$, model results are not highly sensitive to the shape of the assumed onset to detection distribution. Subclinical individuals remain asymptomatic and hence do not receive symptom-triggered testing, although they may be tested and detected as a result of contact tracing (see next paragraph).

In addition to symptom-triggered testing, we assume that a proportion $p_{trace}=0.7$ of infected contacts (whether clinical or subclinical) of a confirmed case are identified via contact tracing and quarantined with a mean of 3 days from confirmation of the index case. In reality, some contacts were scheduled for testing on day 5 and day 12 after exposure and contact management differed for close and casual contacts; however, we do not attempt to model these details of the contact tracing process. We use quarantine to refer to pre-symptomatic or asymptomatic individuals identified via contact tracing who have not yet returned a positive test result, and isolation to refer either to contacts who have developed symptoms or to confirmed cases. We assume that isolation begins either at the time of symptom onset for traced contacts, or at the time of confirmation, whichever is earliest. We assume quarantine reduces transmission by 50% and isolation prevents all further transmission.

## Age-structured transmission model

Transmission between age groups is described by a next generation matrix, whose $\left( i,j \right)$ element is defined to be the expected number of secondary infections in age group $i$ caused by an infected individual in age group $j$ in the absence of control measures and given a fully susceptible population:

$$NGM_{ij}=U\left( p_{clin,j}+\tau(1-p_{clin,j}) \right)u_{i}M_{ji}$$

This formulation models the average number of secondary infections as the product of the average number of contacts an individual in age group $j$ has with someone in age group $i$ during their infectious period ($M_{ji}$), the probability of transmission per contact ($U$), the relative infectiousness of individuals in age group $j$ ($p_{clin,j}+\tau(1-p_{clin,j})$), and the relative susceptibility of individuals in age group $i$ ($u_{i}$). The matrix $M$ describing contact rates between and within age groups was that of (1), which was based on the pre-pandemic estimates of (12) and adapted for New Zealand’s population. The basic reproduction number $R_{0}$ is equal to the dominant eigenvalue of the next generation matrix, denoted $\rho(NGM)$. The constant $U$ represents the intrinsic transmissibility of the virus and the value of $U$ is chosen so that $\rho(NGM)$ is equal to the assumed value of $R_{0}=6$.

The number of people in age group $j$who have had $d$ doses of the vaccine who are infected by clinical individual $l$between time $t$and $t+\delta t$ is a Poisson distributed random variable with mean

$$\lambda_{l,jd}\left( t \right)=Y_{l}C\left( t \right)F_{l}\left( t \right)\left( \int_{t}^{t+\delta t} w\left( t'-t_{inf,l} \right)dt' \right){NGM}_{j,a_{l}}^{clin}\left( 1-e_{Td_{l}} \right)\left( 1-e_{Id} \right)s_{jd}(t)$$

where:

- $Y_{l}$is a gamma distributed random variable with mean 1 and variance $1/k$ representing individual heterogeneity in transmission. We set $k=0.5$ which represents a moderate level of over-dispersion and is consistent with estimates for SARS-CoV-2 transmission patterns (13, 14).
- $C\left( t \right)$ is a time-varying control parameter that is fitted to data.
- $F_{l}\left( t \right)$ represents the effect of quarantine or isolation on the transmission rate of individual $l$at time $t,$ and is equal to 1 if individual $l$is not in quarantine/isolation at time $t$, equal to $c_{quar}=0.5$ if individual $l$ is in quarantine, and equal to $c_{isol}=0$ if individual $l$ is in isolation.
- $w(t')$ is the probability density function of the assumed generation time distribution and $t_{inf,l}$ is the time individual $l$was infected.
- ${NGM}_{j{,a}_{l}}^{clin}=Uu_{j}M_{a_{l},j}$ is the next generation matrix for clinical individuals and $a_{l}$ is the age group of individual $l$.
- $s_{jd}(t)$ is the fraction of age group $j$ that is has not previously been infected and has had $d$ doses of the vaccine at time $t$.

The expression for $\lambda_{l,jd}(t)$ above is multiplied by $\tau$ if individual $l$ is subclinical. Note that the factor $Y_{l}$ means that, in the absence of control measures, the total number of people infected by a randomly selected individual over the course of their infectious period and in a fully susceptible population has a negative binomial distribution with mean $R_{0}$ and variance $R_{0}(1+R_{0}/k)$ (15).

Each daily time step, the susceptible compartment $s_{jd}(t)$ is depleted according to the number of newly infected individuals in that compartment. Prior infection is assumed to provide complete immunity against re-infection for the duration of the model simulation. In addition, a proportion of $s_{jd}(t)$ moves to $s_{j,d+1}(t)$ according to the number of people in age group $j$ who received their $\left( d+1 \right)^{th}$ dose of the vaccine that day.

Under this formulation, all vaccinated individuals have their probability of infection given exposure reduced by $e_{I}.$ This is known as a leaky vaccine model as opposed to an all-or-nothing vaccine model, where a proportion $e_{I}$ of vaccinated individuals are completely immunised and a proportion $1-e_{I}$ are completely susceptible (16). Reality may be somewhere between these idealised models (i.e. there may be some individual heterogeneity in the level of protection provided by the vaccine but not as extreme as all-or-nothing). The all-or-nothing and the leaky vaccine model behave similarly when the proportion of the population with immunity from prior infection is relatively small.

Waning of immunity from either vaccination or from prior infection is ignored. In reality, immunity wanes with time since most recent dose of the vaccine. However, this can be counteracted to a large extent by a third (booster) dose of the vaccine. In New Zealand, a third dose was offered to all adults starting in December 2021, initially with a minimum 6 month interval between the second and third dose. Since we only model the period up to mid January 2022, when the majority of fully vaccinated New Zealanders were still within 3 months of their second dose, the effects of waning are likely to be relatively weak and we do not attempt to model the dynamics of waning and boosting of immunity.

The expected number of secondary infections in age group $i$ caused by an infected individual in age group $j$ in the absence of control measures other than vaccination is (1):

$${NGM}_{ij}^{v}(t)=Uu_{i}M_{ji}\frac{\sum_{d=0}^{2} \left( 1-e_{Id} \right)s_{id} \sum_{d=0}^{2} \left( 1-e_{Id} \right)\left( 1-e_{Td} \right)\left( {\left( 1-e_{Sd} \right)p}_{clin,j}+\tau\left( 1-\left( 1-e_{Sd} \right)p_{clin,j} \right) \right)s_{jd}}{\sum_{d=0}^{2} (1-e_{Id})s_{jd}}$$

The time-varying value of $R_{eff}(t)$ is a model output defined as

$$R_{eff}\left( t \right)=C(t)\left( 1-E(t) \right)\rho\left( NGM^{v}(t) \right)$$

where $\rho(NGM^{v}(t))$ is the dominant eigenvalue of the next generation matrix at time $t$, $C(t)$ is the value of the control function at time $t$, and $E(t)$ is the effectiveness of the test-trace-isolate-quarantine (TTIQ) system in the model in reducing transmission. The value of $E(t)$ is calculated by calculating the average reduction in transmission from the time each case is put into quarantine or isolation.

## Hospitalisation and fatality model

Clinical individuals in age group $i$ with $d$ doses of the vaccine are assumed to require hospitalisation with probability $\left( 1-e_{Dd} \right)/(1-e_{Sd}) p_{hosp,i}/p_{clin,i}$ where $e_{Dd}$ is the vaccine effectiveness against severe disease in breakthrough infections after $d$ doses, and $p_{hosp,i}$ is the infection hospitalisation ratio for unvaccinated people in age group $i$ (see Supplementary Table S1). The time between symptom onset and hospitalisation is assumed to be exponentially distributed with mean 5 days (this assumption affects the timing but not the number of hospital admissions). The length of hospital stay is assumed to be exponentially distributed with mean 8 days (17) (this assumption affects the number of hospital beds occupied at any one time but not the total number of hospital admissions). Hospitalised cases in age group $i$ die with probability $\left( 1-e_{M,d} \right)/\left( 1-e_{D,d} \right)IFR_{i}/p_{hosp,i}$ where $IFR_{i}$ is the infection fatality ratio for unvaccinated cases in age group $i$ (see Supplementary Table S1). For simplicity, the date of death is assumed to be the same as the date of hospital discharge. In reality, the average time from hospital admission to death is longer (this assumption means that deaths will be more lagged relative to cases in reality than in the model but does not affect the total number of deaths).

## Initial conditions

Each simulations was initialised with 200 seed infections with infection times uniformly chosen in the 8 day period from the simulation start date of 10 August 2021. The age groups of seed infections were chosen in proportion to the population age structure. The vaccination status of a seed infection in age group $j$ was chosen according to the probability of a randomly chosen infection in age group $j$ on the simulation start date having either 0, 1 or 2 doses. Any infections that are assigned a confirmation time prior to the time of the first detected case on 17 August 2021 are re-assigned a new confirmation time uniformly chosen at random in the 7 day period following 17 August 2021.

| **Parameter** | | | | | | | | | | | | **Value** | | | | | | |
| --- | --- | --- | --- | --- | --- | --- | --- | --- | --- | --- | --- | --- | --- | --- | --- | --- | --- | --- |
| Basic reproduction number in the absence of control | | | | | | | | | | | | $R_{0}=6$ | | | | | | |
| Incubation period | | | | | | | | | | | | Mean 5.5 days, s.d. 3.3 days | | | | | | |
| Generation interval | | | | | | | | | | | | Mean 5.0 days, s.d. 1.9 days | | | | | | |
| Relative infectiousness of subclinical individuals | | | | | | | | | | | | $\tau=0.5$ | | | | | | |
| Heterogeneity in individual reproduction number | | | | | | | | | | | | $k=0.5$ | | | | | | |
| Vaccine effectiveness:   - against infection (one/two doses) - against symptoms in breakthrough infection (one/two doses) - against transmission in breakthrough infection (one/two doses) - against severe disease in breakthrough infection (one/two doses) | | | | | | | | | | | | $e_{I1}=0.55$ $e_{I2}=0.7$  $e_{S1}=0$ $e_{S2}=0.5$  $e_{T1}=0$ $e_{T2}=0.375$  $e_{D1}=0.6$ $e_{D2}=0.8$ | | | | | | |
| Probability of detection for clinical individuals | | | | | | | | | | | | $p_{test}=0.45$ | | | | | | |
| Probability of a contact of a confirmed case being traced | | | | | | | | | | | | $p_{trace}=0.7$ | | | | | | |
| Relative transmission rate for individuals in quarantine | | | | | | | | | | | | $c_{quar}=0.5$ | | | | | | |
| Relative transmission rate for individuals in isolation | | | | | | | | | | | | $c_{isol}=0$ | | | | | | |
| Time from symptom onset to test result | | | | | | | | | | | | Mean 4.0 days, s.d. 4.0 days | | | | | | |
| Time from confirmation of case to quarantine of contacts | | | | | | | | | | | | Mean 3.0 days, s.d. 1.7 days | | | | | | |
| Time form symptom onset to hospital admission | | | | | | | | | | | | Mean 5.0 days, s.d. 5.0 days | | | | | | |
| Length of hospital stay | | | | | | | | | | | | Mean 8.0 days, s.d. 8.0 days | | | | | | |
| **Age-specific parameters** | | | | | | | | | | | | | | | | | |  |
| Age (yrs) | 0-4 | 5-9 | 10-14 | 15-19 | 20-24 | 25-29 | 30-34 | 35-39 | 40-44 | 45-49 | 50-54 | | 55-59 | 60-64 | 65-69 | 70-74 | 75+ |  |
| Pr(clinical) (%) | 54.4 | 55.5 | 57.7 | 59.9 | 62.0 | 64.0 | 65.9 | 67.7 | 69.5 | 71.2 | 72.7 | | 74.2 | 75.5 | 76.8 | 78.0 | 80.1 |  |
| Pr(hosp)(%) | 0.95 | 0.95 | 0.41 | 0.61 | 0.88 | 1.26 | 1.84 | 2.69 | 3.80 | 5.56 | 8.17 | | 11.4 | 16.2 | 22.2 | 30.0 | 49.0 |  |
| Pr(death)(%) | 0.001 | 0.001 | 0.001 | 0.003 | 0.005 | 0.010 | 0.020 | 0.038 | 0.075 | 0.15 | 0.29 | | 0.56 | 1.10 | 2.10 | 4.00 | 13.5 |  |
| Susceptibility* | 0.46 | 0.46 | 0.45 | 0.56 | 0.80 | 0.93 | 0.97 | 0.98 | 0.94 | 0.93 | 0.94 | | 0.97 | 1.00 | 0.98 | 0.90 | 0.86 |  |
| Popn (1000s) | 306 | 327 | 335 | 315 | 337 | 378 | 380 | 338 | 311 | 328 | 329 | | 326 | 295 | 251 | 217 | 339 |  |

**Supplementary Table S1.** Parameter values used in the model. *Susceptibility for age group $i$ is stated relative to susceptibility for age 60-64 years. Age-dependent rates of clinical disease are based on (18). Pr(hosp) is the age-dependent infection hospitalisation ratio for unvaccinated individuals based on (19) and adjusted by a hazard ratio of 2.26 for the Delta variant (20) and further increased by an odds ratio of 2 for the Auckland outbreak model to provide a better match with hospital admissions data. Pr(death) is the infection fatality ratio for unvaccinated individuals based on (19). Infection hospitalisation ratios and infection fatality ratios vaccinated individuals were reduced according to the relevant vaccine effectiveness parameters, uniformly across all age groups. Age-dependent susceptibility is based on (2). This may partly reflect the elevated risk profile in the subpopulations predominantly affected by the outbreak.

# Supplementary Figures


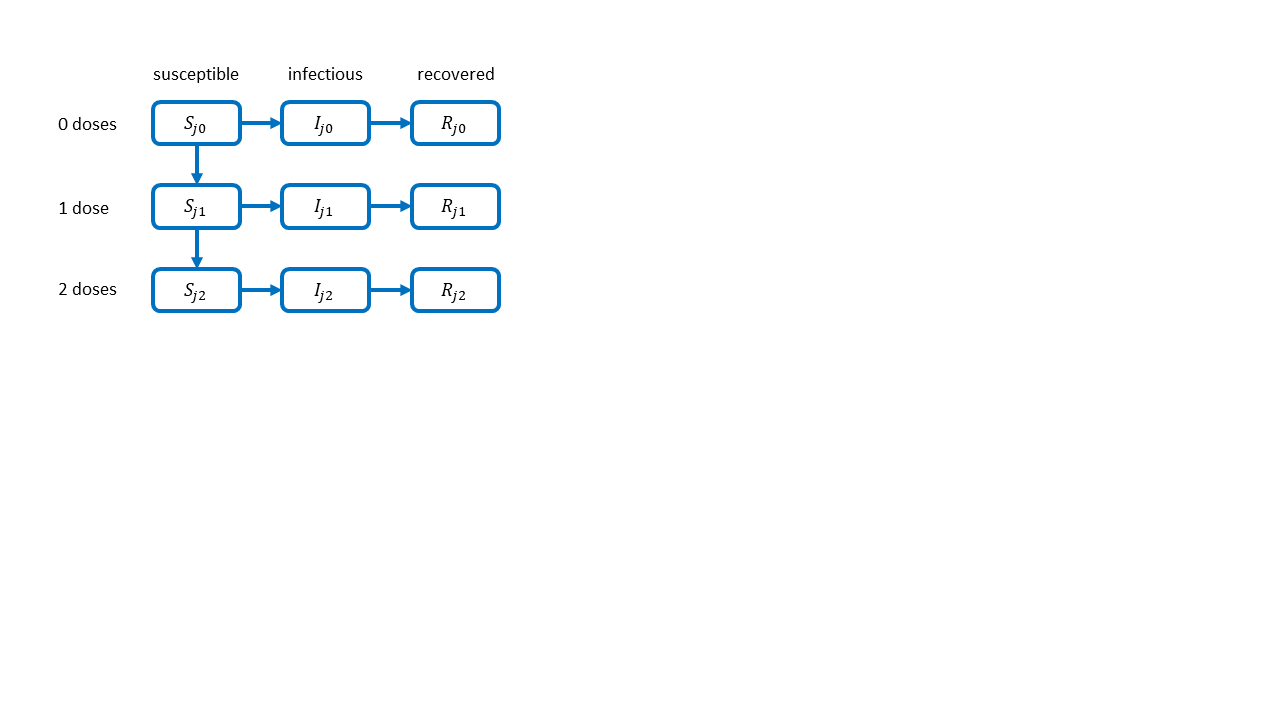


**Supplementary Figure S1.** Diagram showing model compartments for each age group $j$. Individuals progress from $S_{j0}$ to $S_{j1}$ or from $S_{j1}$ to $S_{j2}$ as a result of receiving a vaccine dose. The force of infection on compartments $S_{j1}$ and $S_{j2}$ is reduced by a factor of $e_{I1}$ and $e_{I2}$ respectively relative to compartment $S_{j0}$. In addition, individuals in infectious compartments $I_{j1}$ and $I_{j2}$ have a probability of transmission that is reduced by $e_{T1}$ and $e_{T2}$ respectively relative to compartment$I_{j0}$. Recovered individuals are assumed to remain immune to infection for the duration of the model simulation, regardless of vaccination status.


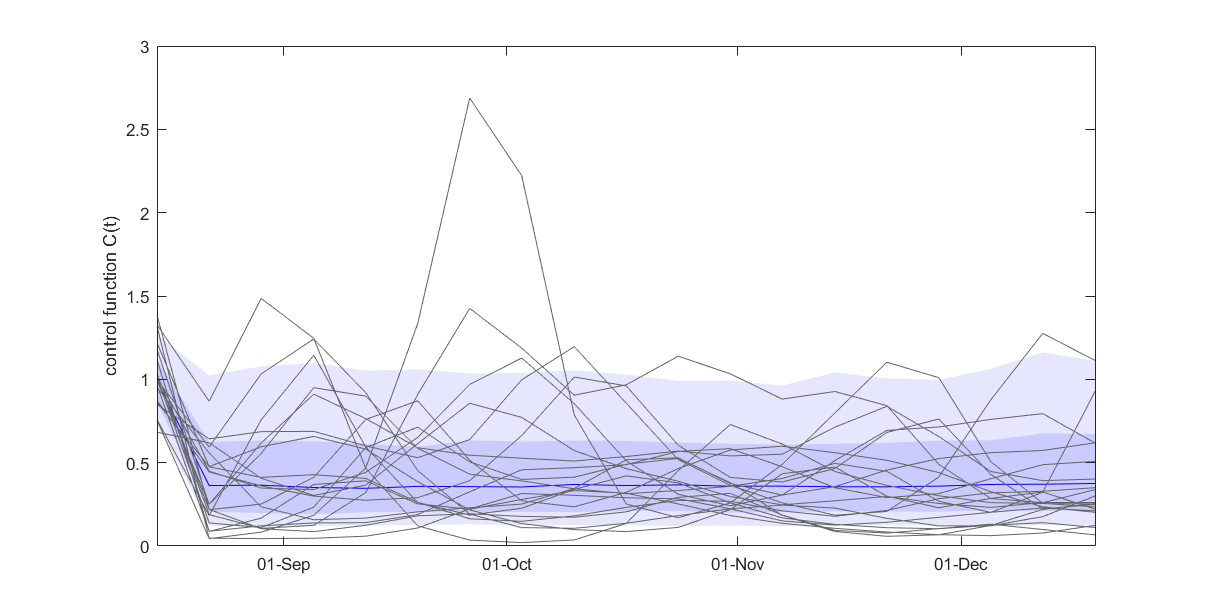


**Supplementary Figure S2.** Prior for the control function $C(t)$ showing the median (solid blue curve), 50% CrI (dark blue shading), 80% CrI (light blue shading), and 50 random draws from the prior (grey curves).


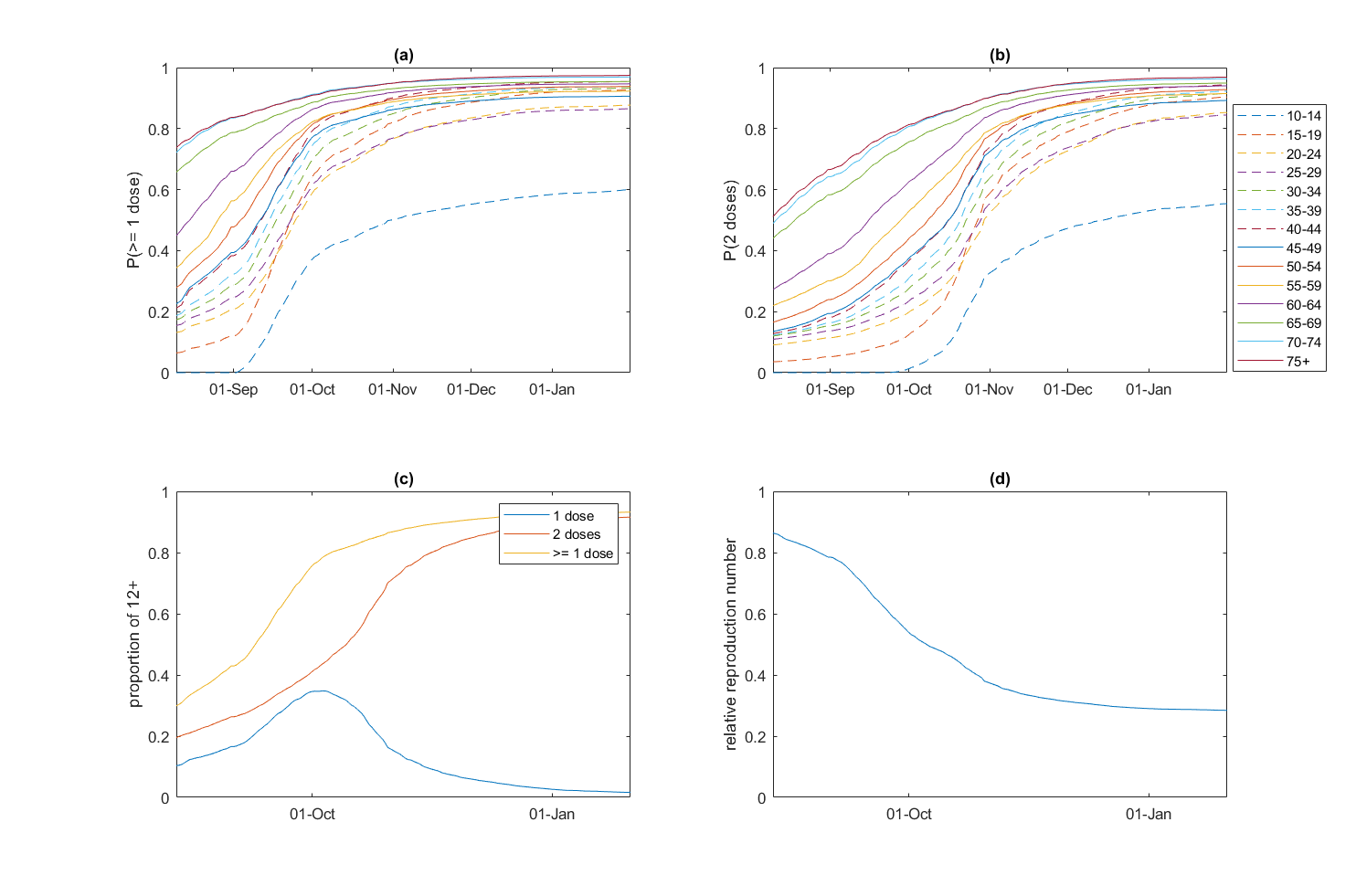


**Supplementary Figure S3.** Proportion of the population in 5 year age bands who have received (a) at least one dose and (b) two doses over time. Results are based on data on vaccines administered up to 7 December 2021, data available on 7 December 2021 on future vaccine bookings, and projected future uptake of second doses.

**References**

1. Steyn N, Plank MJ, Binny RN, Hendy S, Lustig A, Ridings K. A COVID-19 vaccination model for Aotearoa New Zealand. Scientific Reports. 2022;12:2720.

2. Davies NG, Klepac P, Liu Y, Prem K, Jit M, group CC-w, et al. Age-dependent effects in the transmission and control of COVID-19 epidemics. Nature Medicine. 2020;26:1205-11.

3. Byambasuren O, Cardona M, Bell K, Clark J, McLaws M-L, Glasziou P. Estimating the extent of asymptomatic COVID-19 and its potential for community transmission: Systematic review and meta-analysis. Official Journal of the Association of Medical Microbiology and Infectious Disease Canada. 2020;5:223-34.

4. Buitrago-Garcia D, Egli-Gany D, Counotte MJ, Hossmann S, Imeri H, Ipekci AM, et al. Occurrence and transmission potential of asymptomatic and presymptomatic SARS-CoV-2 infections: A living systematic review and meta-analysis. PLoS Medicine. 2020;17(9):e1003346.

5. Lauer SA, Grantz KH, Bi Q, Jones FK, Zheng Q, Meredith HR, et al. The incubation period of coronavirus disease 2019 (COVID-19) from publicly reported confirmed cases: estimation and application. Annals of Internal Medicine. 2020;172(9):577-82.

6. Ferretti L, Wymant C, Kendall M, Zhao L, Nurtay A, Abeler-Dörner L, et al. Quantifying SARS-CoV-2 transmission suggests epidemic control with digital contact tracing. Science. 2020;368(6491).

7. Ryu S, Kim D, Lim J-S, Ali ST, Cowling BJ. Changes in the serial interval and transmission dynamics associated with the SARS-CoV-2 Delta variant in South Korea. Emerging Infectious Diseases. 2021;28(2).

8. Pung R, Mak TM, Kucharski AJ, Lee VJ. Serial intervals in SARS-CoV-2 B. 1.617. 2 variant cases. The Lancet. 2021;398(10303):837-8.

9. Kang M, Xin H, Yuan J, Ali ST, Liang Z, Zhang J, et al. Transmission dynamics and epidemiological characteristics of SARS-CoV-2 Delta variant infections in Guangdong, China, May to June 2021. Eurosurveillance. 2022;27(10):2100815.

10. Zhang M, Xiao J, Deng A, Zhang Y, Zhuang Y, Hu T, et al. Transmission dynamics of an outbreak of the COVID-19 Delta variant B. 1.617. 2—Guangdong Province, China, May–June 2021. China CDC Weekly. 2021;3(27):584-6.

11. Li B, Deng A, Li K, Hu Y, Li Z, Shi Y, et al. Viral infection and transmission in a large, well-traced outbreak caused by the SARS-CoV-2 Delta variant. Nature Communications. 2022;13:460.

12. Prem K, Cook AR, Jit M. Projecting social contact matrices in 152 countries using contact surveys and demographic data. PLoS Computational Biology. 2017;13(9):e1005697.

13. James A, Plank MJ, Hendy S, Binny RN, Lustig A, Steyn N. Model-free estimation of COVID-19 transmission dynamics from a complete outbreak. PLoS ONE. 2020;16:e0238800.

14. Riou J, Althaus CL. Pattern of early human-to-human transmission of Wuhan 2019 novel coronavirus (2019-nCoV), December 2019 to January 2020. Eurosurveillance. 2020;25(4):2000058.

15. Lloyd-Smith JO, Schreiber SJ, Kopp PE, Getz WM. Superspreading and the effect of individual variation on disease emergence. Nature. 2005;438(7066):355-9.

16. Moore S, Hill EM, Tildesley MJ, Dyson L, Keeling MJ. Vaccination and non-pharmaceutical interventions for COVID-19: a mathematical modelling study. The Lancet Infectious Diseases. 2021;21(6):793-802.

17. Steyn N, Binny RN, Hannah K, Hendy S, James A, Lustig A, et al. Māori and Pacific People in New Zealand have higher risk of hospitalisation for COVID-19. New Zealand Medical Journal. 2021;134(1538):28-43.

18. Hinch R, Probert W, Nurtay A, Kendall M, Wymant C, Hall M, et al. Effective configurations of a digital contact tracing app: A report to NHSX. 2020;16 April 2020:29.

19. Herrera-Esposito D, de los Campos G. Age-specific rate of severe and critical SARS-CoV-2 infections estimated with multi-country seroprevalence studies. BMC Infectious Diseases. 2022;22:311.

20. Twohig KA, Nyberg T, Zaidi A, Thelwall S, Sinnathamby MA, Aliabadi S, et al. Hospital admission and emergency care attendance risk for SARS-CoV-2 delta (B. 1.617. 2) compared with alpha (B. 1.1. 7) variants of concern: a cohort study. Lancet Infectious Diseases. 2021;22:35-42.
